# Comparison of remote learning methods to on-site teaching -randomized, controlled trial

**DOI:** 10.1186/s12909-023-04759-3

**Published:** 2023-10-19

**Authors:** Marko Tolonen, Miika Arvonen, Marjo Renko, Heikki Paakkonen, Helena Jäntti, Eija Piippo-Savolainen

**Affiliations:** 1 University of Eastern Finland, Kuopio, Finland; 2 Savonia University of Applied Sciences, Kuopio, Finland; 3 Kuopio University Hospital, Kuopio, Finland; 4 Arcada University of Applied Sciences, Helsinki, Finland

**Keywords:** Vodcast, Podcast, Team-teaching, Streamed teaching, Learning, Remote learning

## Abstract

**Background:**

In the digitalized world, there is a need for developing new online teaching and learning methods. Although audio and video recordings are increasingly used in everyday learning, little scientific evidence is available on the efficacy of new online methods. This randomized trial was set out to compare the learning outcomes of online and classroom teaching methods in training healthcare students to diagnose breathing difficulties in children.

**Methods:**

In total, 301 students of medicine (N = 166) and nursing (N = 135) volunteered to participate in this total sampling study in 2021–2022. The students were randomized into four groups based on teaching methods: classroom teaching (live, N = 72), streamed classroom teaching (live-stream, N = 77), audio recording (podcast, N = 79) and video recording (vodcast, N = 73). Each 45-minute lesson was taught by the same teachers and used the same protocol. The students participated an online test with their own electronic device at three distinct time points: prior to any teaching (baseline), immediately after teaching (final test), and five weeks later (long-term memory test). The test consisted of 10 multiple-choice questions on recognizing breathing difficulties from real-life videos of breathing difficulties in pre-school age. The test results scale ranged from − 26 to 28 points. Statistical analyses were performed using ANOVA multiple comparison and multiple regression tests.

**Results:**

The mean scores (SD) of the final tests were 22.5 (5.3) in the vodcast, 22.9 (6.1) in the live, 20.0 (5.6) in the podcast (p < 0.05 vs. live) and 20.1 (6.8) in the live-stream group. The mean difference of test scores before and after the lesson improved significantly (p < 0.05) in all study groups, with 12.9 (6.5) in the vodcast, 12.6 (5.6) in the live, 10.9 (7.0) in the live-stream and 10.4 (6.9) in the podcast group. The improvement in test scores was significantly higher in the vodcast (p = 0.016) and the live (p = 0.037) groups than in the podcast group. No significant differences were found between the other groups. However, there was a nonsignificant difference towards better results in the vodcast group compared to the live-stream group.

**Conclusions:**

While the new online teaching methods produce learning, only video learning is comparable to team teaching in classrooms.

## Background

The COVID-19 pandemic in 2020 permanently changed learning challenges and opportunities around the world by forcing schools and universities to fully transition to distance learning in just a few months [1–4].

Now, after the pandemic, distance learning methods seem to have become a permanent part of our education also in the fields of health care, medicine, and nursing [5, 6]. However, overall satisfaction with remote teaching has been low, especially among those who are not used to the methods and face challenges in technology competence [7]. In addition, there is considerable variation in the quality and accuracy of freely available online teaching materials, which reveals a need for developing high-quality materials (videos) with scientifically approved content for health care learning [8]. Similarly, there is a lack of controlled studies on the efficacy of distance teaching for producing sufficient learning, especially in clinical diagnostics.

Despite the at least partial shift to distance learning, the principles of learning have not changed. Motivation to learn remains vital and should be promoted, for example by the MUSIC model constructed by Professor Jones in 2016. The model is based on five valid dimensions underlying the student’s motivation: eMpowerment, Usefulness, Success, Interest and Caring [9–11]. Mnemonics have similarly been proven to serve as effective promotors of learning even though they are no longer widely used [12].

In this study, we wanted to promote the identification of breathing problems in children and to study the effectiveness of new distance teaching methods, vodcast and podcast, compared to live and streamed teaching. We chose the topic because learning the mechanism of breathing is multidimensional and complicated in practice and especially remotely [13, 14]. Breathing is affected by several physiological principles [13] with different pathophysiological etiologies causing similar breathing patterns. Tachypnea, for example, can be due to respiratory and metabolic reasons or hyperventilation. Failure to recognise breathing difficulties in time may lead to prolonged and unnecessary suffering, particularly among young children unable to express their symptoms verbally [14].

### Vodcast

As early as the turn of the millennium, it has been shown that video recordings produced with high quality can be useful in learning [15]. Currently, this teaching method utilising video is referred to as a vodcast, which is a video recording that is watchable and listenable on a smart device and thus makes repetitive learning possible. Medical students seem to appreciate the use of videos as supplementary material, but not as a compensatory learning method [16, 17]. In this study, we used the term vodcast, by which we mean a video recording.

Several studies have suggested video recordings to be a higher quality pre-material for on-site learning in comparison with textbooks and conventional online learning methods [17, 18]. However, students have preferred more on-site teaching to video recordings when used as an exclusive teaching method, despite comparable immediate learning results [16].

### Podcast

A podcast is a digitally implemented audio file that can be uploaded to a smart device by the listener or listened to directly from a playback service [19–21].

While podcasts are frequently used in medical education, there is sparse data on the efficacy of podcasts in medical learning. A recent Canadian study found no significant difference in learning by podcast or blog writing (blog) [22], whereas a study from North America argued that surgical nurse students attending traditional classroom lectures scored higher in exams than those who had only listened to prerecorded class lectures through an iPod™ pocket music player device [23]. However, the frequency of using the iPod™ improved the nurse students’ self-reported skills [23].

### Promoting learning by new teaching strategies in classroom teaching: team, activating and multimedia teaching

In addition to new learning methods (vodcast and podcast), on-site team teaching has also received good feedback. In this method, two teachers give a lesson together in the same room. The method has been recently shown to be an efficient way to engage students [24, 25] by making lessons more interesting, stimulating, and informative compared to lessons given by one teacher [24]. Better contact between teachers and students in team teaching lessons results in active conversations on the lesson topic and makes teachers more receptive to critical review [24, 25]. Likewise, activating learners with short exercises and activities during the lesson seems to play a supporting role in learning [26].

Another way to promote long-term learning is to provide information in multiple ways during a single lesson. This sort of multimedia learning involves providing combinations of content in different formats such as text, audio, images, animations, or videos in a single interactive presentation. While this is an effective learning method, it can also overwhelm the learner with cognitive processing requirements. The cognitive theory of multimedia learning (CTML) relies on an understanding of how people learn from words and images, which is particularly important in the context of medical education [24, 25, 27].

### Streamed teaching

Streamed teaching means that the learners follow live classroom teaching via the internet either simultaneously or later using a video recording. This method has gained popularity in recent years, although research data showed controversial results on the effect of the method on students’ academic performance already before the COVID-19 pandemic. An Australian review that included 71 articles (2016) on the learning of high school students showed that the impacts on learning outcomes were not positive. The results were evaluated within four result-efficient dimensions: academic, social, psychological and teacher’s mediation [28].

However, live-streamed learning methods may have some positive outcomes, as evidenced in two Canadian studies. In a study by Grafton-Clarke and colleagues (2022), live streaming was used in medical education in the context of a real-life patient interview situation. The students evaluated the method in terms of quality and found it effective (9.7/ 10 points) [29]. Another Canadian study found that an innovative (POV = point of view) live-streaming tool was more effective compared to video learning [30].

### Objectives

The purpose of the present study was to compare the learning results between online and classroom teaching methods in teaching health care students to identify and treat breathing difficulties in children.

In this study, we wanted to combine team teaching with the new methods and create modern vodcasts, podcasts and activating lessons, and to compare their effectiveness. We chose a child’s respiratory distress lesson for our approach as it is a challenging task even for experienced physicians [14, 31]. Identifying a child’s breathing difficulties requires repeated learning that challenges educational resources.

As far as we know, there are no studies in the medical education field comparing vodcast, podcast and live-stream didactics as learning methods, at least in the context of learning to identify respiratory distress in child patients. Thus, we deemed it relevant to compare the efficacy and experience of such learning methods for the identification of acute respiratory distress in children.

### Study design

The study consisted of eight separate teaching events organised during the academic year 2021–2022. Each event included 40 students randomly assigned to four small groups (n = 10) that received teaching by different methods: teaching in the classroom (live), streamed classroom teaching (live-stream), audio recording (podcast) and video recording (vodcast). Each student participated in the lesson only once.

**Fig. 1:**
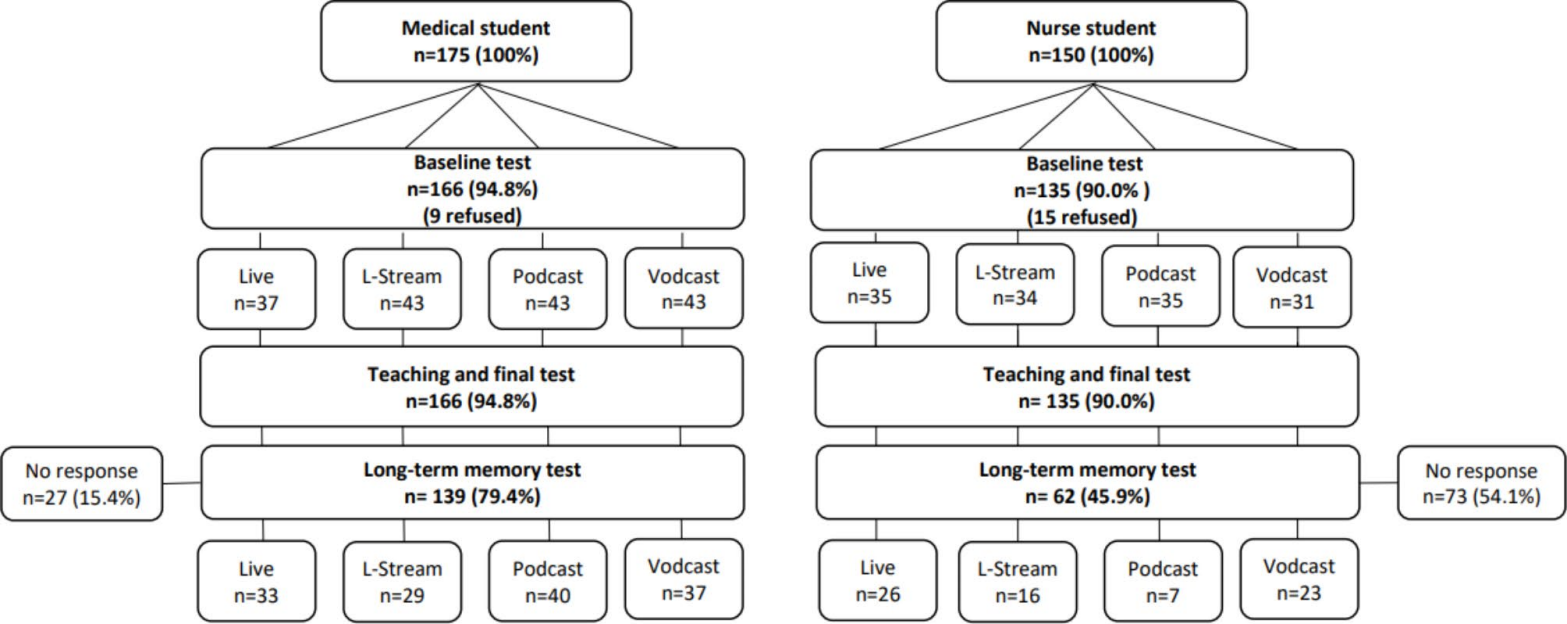
Study design

The study design and participant flow are described in Fig. 1. Prior to the lesson, all of the participants took a baseline test measuring their knowledge of respiratory distress in children. After the test, teaching was performed simultaneously in the four teaching groups. At the end of the lesson, each group retook the same test (the final test). The total duration of the baseline test, teaching event and final test was 75 min. Five to seven weeks later, long-term learning was evaluated by repeating the test once more.

### Participants and methods

#### Participants

A total of 325 students (175 medical students, 150 nurse students) were recruited to participate in the trial. All medical students were in their fifth year of studies at the University of Eastern Finland and participated in an eight-week paediatric course implemented between August 2021 and May 2022. Additionally, paramedic nurse (annual courses from one to four) students from the Savonia University of Applied Sciences in Kuopio (Finland) participated in the study during their paediatric course at the same time as the medical students. Both medical and nurse student groups were invited to participate in this randomized controlled trial. In this study, we refer to these professional groups as medical students and nurse students.

The participants were randomized into four groups: classroom teaching, streamed teaching, podcast and vodcast groups (Fig. 1.). A total of 24 students taking the baseline test did not give their permission to participate in this research or their data could not be combined at the analysis stage based on their identification codes. The participation rate was 93%.

All fifth-year medical students (n = 175) and all nurse students from each of the four classes (n = 150) were asked to participate in the study. Participation in the study was voluntary, but the examined teaching was a part of the medical students’ curriculum concerning respiratory distress in children.

#### Sample size calculation and randomization

A pilot study was carried out among 20 paramedic nurse students to estimate baseline mean values and standard deviations in the classroom teaching, podcast or vodcast groups. For sample size calculation (https://clincalc.com), the change in the test scores from baseline to final test was considered as the main outcome and a difference of 1.5 points between the groups was identified as an important difference. With this assumption and α-error of 0.05 and power (1- β) of 0.80, the result of the sample size calculation was 28 students per group.

The percentage of dropouts was estimated to be 30–40% after both the baseline and final tests, resulting in a need to recruit 40 students for each teaching group.

The students were randomized into four equally sized teaching method groups using a computer program (https://www.randomizer.org).

#### Intervention

The teaching took place simultaneously in four teaching groups. The students in the classroom teaching group (n = 72) participated in an on-site class facilitated by the teachers. The students in the remote teaching groups participated in the teaching intervention at their chosen locations using their own computers or smart devices. The podcast (n = 79) and vodcast (n = 73) groups participated in the teaching activity by listening to or watching recorded material. The stream teaching group (n = 77) viewed a live-taught course through Zoom webconferencing software, and had an opportunity to communicate with the teachers, either by speaking through their computer’s microphone or by texting their comments in Zoom’s chat feature.The the podcast and vodcast groups did not have such an opportunity.

The same script and protocol were used in all the teaching groups. The script was jointly prepared by three clinical teachers in paediatrics and evaluated before recording the material. For example, the vodcast was recorded and edited beforehand by adding subtitles. Subsequently, the audio file was isolated from the vodcast into a separate recording, which was subsequently made into a podcast. Therefore, the vodcast and the podcast were the exact same recording with the exception that there was no video content included in the podcast.

**Fig. 2:**
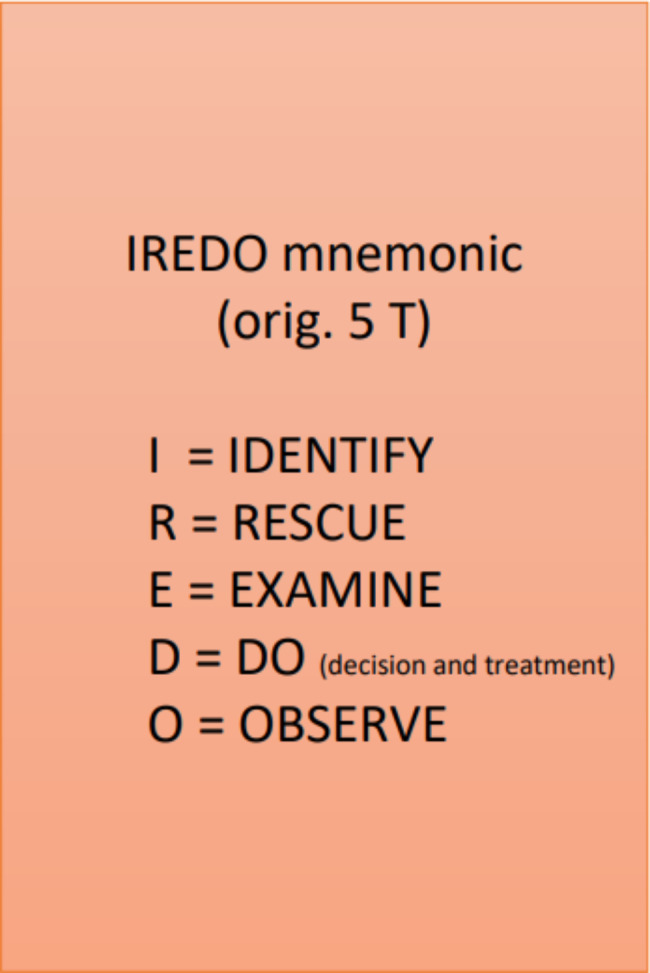
IREDO mnemonic

In all the teaching groups, the lesson was based on the MUSIC (empowerment, usefulness, success, interesting, caring) theory and followed the principles of multimedia teaching [9–11] to promote the students’ motivation and learning. The lesson content was designed to be simple, useful, and interesting by using several ways of teaching, such as sharing real-life experiences, producing typical wheezing sounds, showing videos of children with breathing difficulties, and solving patient cases together. The lesson was performed by two teachers (team teaching) who actively encouraged the students to discuss and complete practical exercises (activating and interactive teaching). The same real-life videos of children with breathing problems were shown in the live, live-stream and vodcast groups; meanwhile, the podcast group had access to only the teacher’s voice describing the findings obtained from the videos. In addition, we constructed a mnemonic (IREDO) on how to act if suspecting breathing difficulties in small children and taught it to all the students (Fig. 2.)

### Content of teaching

The content of the lesson was similar in all the teaching groups and consisted of the identification and medical treatment of the most common respiratory problems in children (Table 1.).

**Table 1:** The content checklist and implementation plan for an activating 45-minute team teaching multimedia learning lesson

1.	The difference between breathing difficulty and respiratory failure (discussion)
2.	The red flags for respiratory distress in children (speech)
3.	IREDO mnemonic: identify, rescue, examination, do decision and treatment, observe. (writing)
4.	A true story of laryngitis and the importance of its prompt treatment (discussion between the teachers)
5.	Inhalation and exhalation difficulties by breathing through straws (demonstration and experience by students)
6.	Differential diagnostics of laryngitis and epiglottitis (speech)
7.	Other causes of inhalation difficulties (speech)
8.	Definition of breathing difficulties by clinical examination and identification of respiratory distress: laryngitis, bronchiolitis and osbtructive bronchitis (real-life videos and power point slides)
9.	Bronchiolitis and obstructive bronchitis (power point slides + speech)
10.	Definition of breathing difficulties by clinical examination (speech)
11.	Wheezing and crackles. (students draw a line describing the typical sound)
12.	High frequency wheezing vs. low frequency wheezing (demonstration)
13.	Fine and coarse crackles (speech)
14.	Pathophysiology of crackles (discussion with students)
15.	The causes of silent lung sounds, light test in pneumothorax (discussion with students)
16.	Breathing difficulty with no inspiratory or expiratory component (discussion with students)
17.	Diabetic ketoacidosis and Kussmaul breathing (speech)
18.	Cheyne-stokes breathing. (speech)
19.	Pews (Pediatric Early Warning Score) criteria and limit values for age (speech)
20.	Specific features of breathing difficulties in neonates. (speech)

### Measure of learning

The primary outcome was how much the learning results improved in the identification of breathing problems in children. Learning was measured using the same Webropol e-Test that was sent to the participants via e-mail on three separate occasions (baseline, final and long-term memory tests). The test was time-constrained (15 min) and consisted of 10 multiple-choice questions: five theoretical questions and five video clips of children with respiratory distress. The number of correct response alternatives was one or more in each question: Every correct choice accounted for plus 2 points and every wrong choice for 1 minus point. The maximum score was plus 28 points, and the minimum was minus 26 points.

Learning efficacy was defined as the difference between the final and baseline test scores.

### Statistical analyses

The IBM SPSS statistical software 27.0 was used to compare and analyze the results of the study.

As the test scores followed the normal distribution, the ANOVA multiple comparison test was used for assessing differences between teaching groups, and post hoc tests for paired comparison were performed using the Least Significant Difference (LSD) test. Multiple regression analysis was used to test the effect of background variables on the results. The level of statistical significance was set at p < 0.05.

### Ethical considerations

All fifth-year medical students (UEF) and all paramedic nurse students (Savonia) were invited to participate in the study.

At the outset of the baseline test, students were requested to provide their consent for participation in the study. The students had the right to participate in each of the teaching interventions even if they opted out of participating in the tests of the present study. The data were collected with a pseudo-descriptive method. The data were collected and analyzed anonymously.

The research plan was evaluated by the Research Ethical Committee of UEF, although its authorization was not required under Finnish law.

## Results

A total of 301 (92.6%) of the invited 325 students participated in the study, including 176 (58.3%) women and 125 (41.7%) men. There were 166 medical students and 135 nurse students, and 50 participants had completed previous education in health care (Table 2.).

**Table 2:** Characteristics of the 301 participants divided by teaching groups

	Live n = 72 (23.9%)	L-Stream n = 77 (25.6%)	Podcast n = 79 (26.2%)	Vodcast n = 73 (24.3%)	Total n = 301 (100%)
Female	45 (62.5%)	46 (59.7%)	44 (55.7%)	41 (55.4%)	176 (58.3%)
Male	27 (37.5%)	31 (40.3%)	35 (44.3%)	32 (44.6%)	125 (41.7%)
Medical students	37 (51.4%)	43 (55.8%)	44 (55.7%)	42 (58.1%)	166 (55.1%)
Nurse students	35 (48.6%)	34 (44.2%)	35 (44.3%)	31 (41.9%)	135 (44.9%)
No prev. degree in health care	64 (88.9%)	60 (77.9%)	64 (81.0%)	63 (86.5%)	251 (83.4%)
Previous degree in health care	8 (11.1%)	17 (22.1%)	15 (19.0%)	10 (13.5%)	50 (16.6%)

Live = classroom teaching, L-stream = online-streamed teaching

Podcast = audio recording, Vodcast = video recording

The results of the baseline tests, presented as mean (SD), were 9.5 in the vodcast (5.8), 10.25 in the live (6.1), 9.6 in the podcast (6.0) and 9.1 in the live-stream (4.9) groups (non-significant). The mean scores (SD) in the final tests were: 22.5 (5.3) in vodcast, 22.9 (3.7) in live, 20.0 (5.6) in podcast (p < 0.05 vs. live) and 20.1 (6.8) in live-stream groups (Table 3.).

**Table 3:** The learning results in each teaching group

Test	Test scores^1^ Mean (SD). Min-max				
	**Live** n = 72	** L-Stream** n = 77	**Podcast** n = 79	**Vodcast** n = 73	**Total** 301 (100%)
Baseline	10.25 (6.1) -4-24	9.1 (4.9) -3-21	9.6 (6.0) -3-26	9.5 (5.8) -3-26	301 (100%)
Final **	22.9 (3.7) 13–28	20.1 (6.8) -3-28	20.0 (5.6)* 5–28	22.5 (5.3) 6–28	301 (100%)
Long-term memory**	22.3 (5.2) 7–28	20.2 (7.3) 2–28	22.6 (4.5) 9–28	22.3 (6.1) 7–28	211 (71%)

^1^ theoretical min − 23 and max 28

*p < 0.05 vs. Live

**p < 0.05 vs. baseline scores in every study group

Live = classroom teaching, L-stream = online streamed teaching

Podcast = audio recording, Vodcast = video recording

The difference in the test scores before and after the lesson improved significantly (p < 0.05) in all the study groups and was 12.9 (6.5) in the vodcast, 12.6 (5.6) in the live teaching session, 10.9 (7.0) in the live-stream and 10.4 (6.9) in the podcast groups (Table 3 and 4). In a comparison of the long-term memory test results between the groups using different learning methods, no statistically significant differences were observed between the four learning methods (n = 211).

There was a statistically significant difference in the final test scores between the methods, favouring the vodcast method compared with the podcast method (p = 0.016). In addition, a significant difference was confirmed between attending live teaching session vs. listening to the podcast (p = 0.037). There was also a trend indicating that the students preferred vodcasts compared to streamed teaching (p = 0.055) (Table 3.).

**Table 4:** Learning efficacy in the four teaching groups

Tests	Score difference (learning efficacy) between the tests^1^. Mean (SD) Min–max				
	**Live** n = 72	** L-Stream** n = 77	**Podcast** n = 79	**Vodcast** n = 73	**Total n** n = 301
Final-baseline	12.6 (5.6)	10.9 (7.0)	10.4 (6.9)*	12.9 (6.5)	301 (100%)
Long-term memory-baseline	11.6 (6.5)	11.0 (6.2)	11.9 (6.0)	12.9 (7.2)	211 (71%)

^1^ theoretical min − 23 and max 28

*p < 0.05 vs. Live

Live = classroom teaching, L-stream = online streamed teaching

Podcast = audio recording, Vodcast = video recording

There were no significant differences in learning outcomes between women and men. The medical students showed significantly better scores than paramedic-nurse students at each of the measurement points. Students with a previous health care degree performed significantly worse in both the final and the long-term memory tests when compared to those without previous education in health care (Table 5.).

**Table 5:** Learning results in the four teaching groups 5–7 weeks after the lesson

Field of study	Test scores, mean, (SD)			
	**Live**	**L-stream**	**Podcast**	**Vodcast**
Medical students n = 139	n = 33 25.3 (2.8)	n = 29 23.8 (4.9)	n = 40 23.7 (3.5)	n = 37 25.5 (2.8)*
Nurse students n = 62	n = 26 18.4 (4.9)**	n = 16 13.7 (6.6)	n = 7 16.0 (4.7)	n = 23 17.1 (6.4)

*p < 0.05 vs. pod, p = 0.07 vs. stream

**p < 0.05 vs. stream

Live = classroom teaching, L-stream = online streamed teaching

Podcast = audio recording, Vodcast = video recording

In connection with educational success at the long-term memory phase, it is noted that medical students were more eager than nurse students to participate in the test regardless of their gender or previous education (Table 6.). Among the nurse students, those who participated in the live classroom teaching and vodcast groups were the most motivated to participate in the long-term memory test (Table 6.).

**Table 6:** Predictive factors for test results in multivariate analysis

Participant characteristics	Test scrores^1^, mean (SD) scale, min- max		
	**Baseline test** n = 301	**Final test** n = 301	**Long memory test** n = 211
Female (n = 176)	10.6 (5.8) -3-26	22.3 (4.7) 6–28	22.0 (6.2) 2–28
Male (n = 125)	9.1 (4.9) -3-21	21.8 (5.0) 6–28	21.8 (7.3) 2–28
Medical student (n = 166)	11.4 (5.6) -3-26*	23.4 (4.1) 6–28*	24.6 (3.6) 5–28*
Nurse student (n = 135)	7.3 (5.3) -4-22	19.6 (5.1) 6–28	16.7 (6.0) 2–28
No previous education (n = 251)	10.1 (6.0) -4-26	22.4 (4.5) 6–28**	22.5 (5.5) 2–28**
Previous education (n = 50)	9.0(4.4) 1–17	16.9 (6.2) 6–28	16.7 (6.1) 4–28

^1^ theoretical min − 23 and max 28 (points)

*p < 0.05 vs. nurse students

**p < 0.05 vs. students with previous education

## Discussion

### Key results

The results of the present study show that the students of different genders, both medical and paramedic nurse students as well as those with or without previous health care education were able to learn with all the examined methods. Among the online teaching methods, only the vodcast was comparable to classroom teaching. The podcast and live-streaming produced poorer learning outcomes.

### Live and vodcast

In our study, learning with the live and vodcast methods was equally effective in line with Schreiber’s observations in 2010. In that randomized study, 100 undergraduate medical students either attended a live lecture or watched a recording of the lecture afterwards once or multiple times. Although no significant difference was found in the students’ test scores immediately after the teaching, they preferred the live session and evaluated it as more engaging than the vodcast. Our study differed from this setting in that the vodcast and podcast in our study were prerecorded, containing content similar to that used with the live and streamed teaching groups with no possibility to watch or listen to the material repeatedly [16]. In addition, the test protocols were different in these two studies: In Schreiber’s research, conclusions were drawn from 34 multiple-choice questions without videos, whereas our test consisted of 10 multiple-choice questions including a transfer test of five respiratory distress videos [25].

Several other studies also support our findings: a study conducted in Ireland (n = 73) demonstrated that access to vodcasts prior to and after traditional medical classroom teaching promotes both short-term and long-term learning [18]. Similarly, in a Canadian study (n = 35) by Narula, health care trainees estimated 5-minute teaching videos to be more effective, appropriate and time-efficient than textbooks and conventional online resources in preparing for patient contacts [17]. In 2016, Guy and Marquis [32] showed that the grades of 433 College Business students were not influenced by the used learning method, neither traditional education nor new recording methods (vodcast and podcast), during a three-year follow-up period. In a recent study, video teaching was also demonstrated to be as effective as live teaching in teaching manual skills to medical students [33, 34].

It should be emphasized that while the vodcast group had good efficacy in our study, this finding cannot be directly applied to video learning via YouTube. The quality and accuracy of videos used to teach medicine on YouTube varies, which makes it necessary to develop high-quality videos with scientifically approved content for health care learning [8]. Our experienced team used 20 h for preparing the 45-minute vodcast.

### Team teaching

A Belgian review of 33 peer-reviewed research articles shows that team teaching supports teachers’ ability to achieve better learning outcomes in cooperation with teachers and learners. Team teaching can create “rich and varied lessons” appreciated by students [35]. In our study, we wanted to share our expertise and to respond better to learners’ needs with team teaching. In teaching, we stressed Richard Mayers’ (2010) approach in the context of children’s respiratory distress videos used in the lessons, which we felt would improve long-term learning.

Effective medical education is underpinned by an understanding of the efficacy of various visual and auditory inputs that form the training material. Retention and transfer tests are used to measure students’ recall and practical application skills post-learning. Mayer has studied the cognitive theory of multimedia learning (CTML) and concluded that information provided via multimedia strengthens long-term learning. In planning teaching, care is required to ensure the learners’ cognitive ability is not overwhelmed by cognitive processing requirements [24, 25].

### Podcast and live stream

Our research is one of the first randomized controlled trials where a podcast is compared to other teaching methods. Some recent studies have suggested that podcasts could be as effective as other e-learning tools [36, 37], such as blog post learning [22], in improving manual skills in medical education [38]. However, the results are not comparable to ours because the teaching has often included additional e-learning materials, such as PowerPoint slides and text, while only an audio recording was available in our study.

Although students tend to prefer prerecorded short podcasts that they can listen to during their daily activities to traditional classroom lessons and express the effectiveness of the method on their skills, their learning grades do not seem to reflect this when compared to on-site teaching [23]. In our study, the students listened to the podcast only once, and we had no information on what the participants did while listening to the podcast.

Our well-prepared podcast seemed to promote effective learning with results equal to streamed teaching. Even though the results were inferior to live teaching or vodcasts, the effectiveness was unexpectedly good, given that the participants did not see the picture and videos of breathing difficulties at all. Maybe using only one sense improves concentration and the ability to absorb details from the material.

Live streaming is a currently widely used teaching method [39], but our results did not support its usefulness. Streamed teaching was less effective than a simultaneous live lesson despite our efforts to activate the remote students through targeted questions, practical exercises, and encouragement to interact verbally or by commenting in a chat feature. Thus, we agree with the results shown in an Australian review from 2016 [28] that pointed out that streamed teaching is not an optimal tool for learning. As our study data was collected during the COVID-19 pandemic (autumn 2021 and spring 2022), the results may have been affected by frustration with distance learning felt by some of the students.

### Long-term learning

When evaluating the effect each method had on long-term learning, we observed a difference in the learning outcomes during the five to seven–week follow-up period among the medical and nurse students. Among the nurses, the scores of the long-term memory test were lower than in the final test in all the teaching groups. In contrast, among medical students, the learning results improved in all groups from the final test to the long-term memory test. This was most likely due to additional teaching (2 h) of breathing difficulties during the course, enabling the students to observe children’s breathing in real life and from video recordings.

Interestingly, the live teaching and video recording groups still tended to get better learning results than the live-stream and podcast groups, despite the provided additional learning for all the medical student groups. The results suggest that live-streamed teaching is not a preferable teaching method if there are other options available.

The results may have been affected by a large number (one-third) of dropouts after the final test. The number of respondents decreased most in the live-stream (42%) and podcast groups (41%). Learning results were also poorer in these groups. The decrease in participation rates in the long-memory test was more evident among the nurse students (54%) than among the medical students (16%).

Our lesson was based on team teaching, socio-constructivism [40] and multimedia learning [25]. It involved the participants’ active participation by drawing the sound of wheezing (continuous line) and crackles (discontinuous line), and mimicking breathlessness by breathing through straws.

As studies on multimedia [24, 25] learning show, there were also elements, such as the videos, mnemonics and cognitive actions during the lesson, in our research that might have contributed to long-term learning. Although our study did not include analyses on these elements, we believe that they promoted the students’ ability to identify and diagnose breathing difficulty also in the long term.

### Strengths

The strengths of our study were a randomized design (RCT), a sufficient number of participants based on power calculation and a high participation rate. The test used to measure learning was sufficiently difficult and the results were statistically normally distributed. We also managed to follow a 20-principle approach to all teaching, so the methods and settings were comparable with each other. Our teaching was in line with the principles of meaningful learning [24, 25] and the recently presented MUSIC model [10]. The statistical analyses were reliably performed under the guidance of the hospital’s biostatistician.

### Weaknesses

The large number of dropouts in the long-term memory test for nurses may have affected the comparison of the teaching groups and reduced the accuracy of statistical analyses.

Even though we managed to follow the 20 principles programme of teaching, the live lessons may have been somewhat different on each occasion due to a varying social atmosphere and learner activity. The long-term learning of the medical students was affected by 2 h of additional training they received on breathing difficulties, which affected the results of the long-term memory test.

## Conclusion

Both activating team teaching and well-prepared pre-recorded video teaching (vodcast) promote learning effectively. Podcasts also promote learning, but they are not as efficient as the aforementioned methods (live and vodcast). In our comparison, the live-stream method of teaching was found to be the weakest learning method and using it should be therefore carefully considered.

## Data Availability

All datasets are presented in the main manuscript.
